# P-25. Evaluating the Immunogenicity and Safety of Either Two High-Dose or Two Standard-Dose Influenza Vaccines Over Two Consecutive Seasons in Adult Hematopoietic Cell Transplant Recipients

**DOI:** 10.1093/ofid/ofae631.232

**Published:** 2025-01-29

**Authors:** Haya Hayek, Einas Batarseh, Tess Stopczynski, Lora D Thomas, Lubna Hamdan, Daniel Dulek, Zaid Haddadin, Justin Z Amarin, Olla Hamdan, Yasmeen Z Qwaider, Laura S Stewart, Edgar T Overton, Michael Ison, Steven A Pergam, Andrew J Spieker, Natasha B Halasa

**Affiliations:** Vanderbilt University Medical Center, Nashville, Tennessee; Vanderbilt University Medical Center, Nashville, Tennessee; Vanderbilt University Medical Center, Nashville, Tennessee; Virginia Commonwealth University, Richmond, Virginia; Vanderbilt University Medical Center, Nashville, Tennessee; Vanderbilt University Medical Center, Nashville, Tennessee; Vanderbilt University Medical Center, Nashville, Tennessee; Vanderbilt University Medical Center, Nashville, Tennessee; Vanderbilt University Medical Center, Nashville, Tennessee; Vanderbilt University Medical Center, Nashville, Tennessee; Vanderbilt University Medical Center, Nashville, Tennessee; University of Alabama at Birmingham, Birmingham, AL; Respiratory Diseases Branch, DMID/NIAID/NIH, Derwood, MD; Fred Hutchinson Cancer Research Center, Seattle, Washington; Vanderbilt University Medical Center, Nashville, Tennessee; Vanderbilt University Medical Center, Nashville, Tennessee

## Abstract

**Background:**

Adult hematopoietic cell transplant (HCT) recipients are at high risk of influenza complications but have suboptimal immune responses to influenza vaccines. Our previous phase II trial in adult HCT recipients demonstrated that two doses of high-dose trivalent influenza vaccine (HD-TIV) were associated with higher hemagglutination inhibition (HAI) antibody responses than two doses of standard-dose quadrivalent influenza vaccine (SD-QIV), with similar safety profiles. This sub-study aims to characterize immunogenicity and safety of this vaccination strategy in consecutive influenza seasons.

**Figure:**
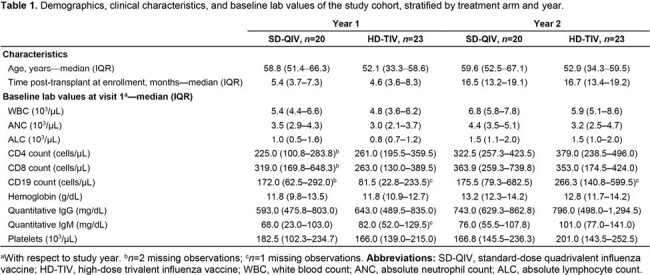

**Methods:**

A subset of participants from our initial study were re-enrolled in a consecutive season using the same dosing regimen. HAI titers were measured at baseline and 4–6 weeks post-vaccination for each dose in both years. We calculated the geometric mean fold rise (GMFR) in HAI titers and used linear mixed-effects models to determine adjusted geometric mean ratios (aGMRs). Injection-site and systemic reactions were documented for 7 days post-vaccination.

**Figure:**
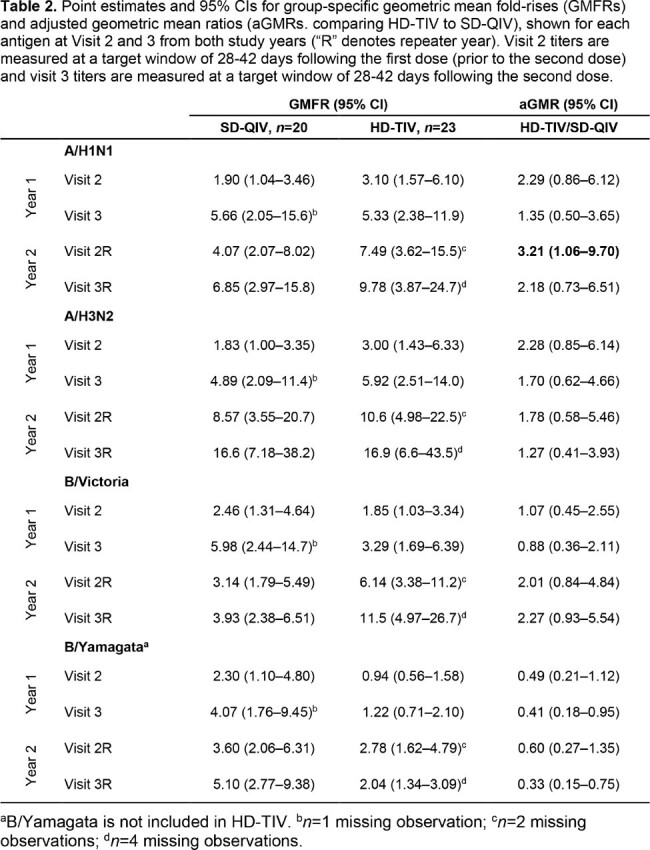

**Results:**

A total of 43 participants were re-enrolled (*n*=20 for SD-QIV and *n*=23 for HD-TIV). Baseline characteristics were similar between groups (**Table 1**). In the second year, post-dose 1 GMFRs for both SD-QIV and HD-TIV were higher compared to post-dose 1 titers in the first year across all antigens (**Table 2**). HAI titers were higher for all post-vaccination visits and dose groups in the second year compared to the first year (**Figure 1**). aGMR comparisons between HD-TIV and SD-QIV favored HD-TIV for A/H1N1, A/H3N2, and B/Victoria antigens in both years, with statistical significance for A/H1N1 after a single dose in the second year (aGMR, 3.21; 95% CI,1.06–9.70). Injection-site and systemic reactions were generally similar between groups in year 2 (**Figure 2**).

**Figure 1.**
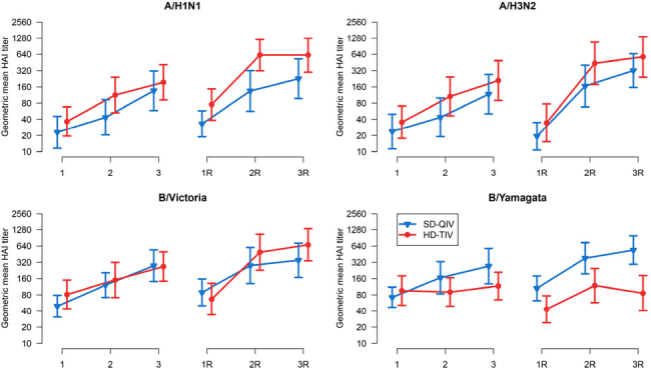
Point estimates and 95% confidence intervals for geometric mean HAI titers at visits 1, 2, and 3 in year 1, as well as visits 1, 2, and 3 in the repeater year (denoted by “R”), stratified by influenza antigens (A/H1N1, A/H3N2, B/Victoria, and B/Yamagata), and by dose group (standard-dose quadrivalent influenza vaccine [SD-QIV] and high-dose trivalent influenza vaccine [HD-TIV]). Note that B/Yamagata is not included in HD-TIV.

**Conclusion:**

In the second year, a single dose of HD-TIV or SD-QIV was more immunogenic than two doses of the same formulation in the first year, with HD-TIV demonstrating superior immunogenicity against A/H1N1. The safety profile across both vaccine formulations were similar.

**Figure 2.**
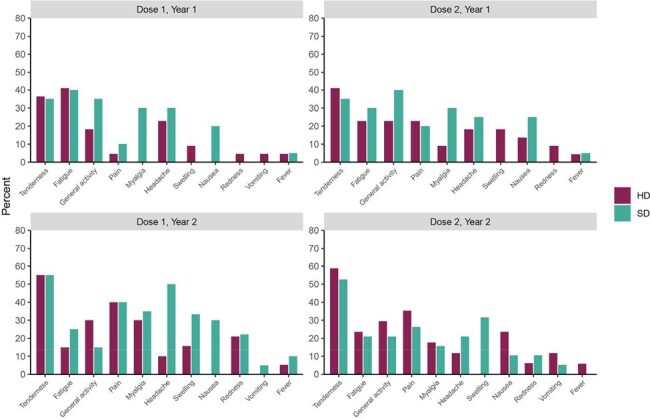
Relative frequencies of any injection-site and systemic reactions within 7 days of each vaccination for both vaccine groups (standard-dose quadrivalent influenza vaccine [SD-QIV] vs high-dose trivalent influenza vaccine [HD-TIV]) following each vaccine dose.

**Disclosures:**

**Edgar T. Overton, MD**, ViiV Healthcare: Employment **Michael Ison, MD MS**, GlaxoSmithKline: Grant/Research Support|UpToDate: Royalties **Steven A. Pergam, MD, MPH**, Cidara: Participate in company sponsored clinical trial|F2G: Participate in company sponsored clinical trial|Global Life Technologies: Grant/Research Support|Symbio: Participate in company sponsored clinical trial **Natasha B. Halasa, MD, MPH**, Merck: Grant/Research Support

